# Memorials to Hospital Worthies

**Published:** 1917-10-27

**Authors:** 


### The Hospital

The Workers' Newspaper of Administrative Medicine and Institutional Life, Administration, National Insurance and Health.

No. 1638, Vol. LXIII. SATURDAY, OCTOBER 27, 1917. PRICE ONE PENNY

### MEMORIALS TO HOSPITAL WORTHIES.

How few so-called memorials to hospital workers, who have rendered material service to institutions with which they have associated themselves, and to the cities and districts where their life's work has been done, however sound may have been the feeling which inspired them, have proved to possess in fact real worth and to be carried through to the end with continuous cordiality! It would be easy to emphasise the serious defects which have attached to memorials, by instances within recent memory, some of which are associated with some of the best of our workers, who will go down to history as magnificent examples of the finest voluntary work done for its own sake and the sake of the standard of life which each worker adhered to throughout life. The reason has been that far too often a memorial of this kind has fallen into the hands of people who have had axes to grind, and so the known wishes of the persons memorialised and their special work, even for an institution which may be immensely indebted to it for much of its success, have come to be disregarded by the substitution of some object which it was the ambition of a few to advance and put through. A lifelong experience, and the pain caused by being associated with and finding oneself powerless to control, or prevent the misuse of such memorial movements, which ought to rest wholly upon the highest motives and the deepest appreciation of the dead worker, whereas this misuse has tended on occasion to threaten the reputation created by sound work and devotion during life, has convinced us that the living have no right to exploit the memory of the dead. Indeed, it would be far better to let them rest in peace without any miscalled memorial of the types in question.

Take the case of a Memorial Committee to which all sorts of people give their names, but to which few or none of them from the outset devote energy and effort to make it worthy and successful. Take again the case of a few people more or less connected with a particular institution with which a deceased worker may have been closely associated, who want to get something done for which money is needed and is not forthcoming, but which has little or no relation to the departed. Or again, take the case of a successor to some man in a public position who feels it incumbent upon him to start a memorial to his predecessor, but who has neither the knowledge, capacity, nor the power to make it a success, and so the movement dies out without result. Examples of each of these have occurred in the last quarter of a century, and we mention them in the hope that nothing of the kind will ever occur again in this country, especially after the war which is having such inspiriting results. Thus, the awakening of our fighting men, with all who are dear to them, the devotion of non-combatant workers of all types who are giving everything of the best that they possess in association with our warriors, in the many campaigns our nation is conducting at the present time, and the heightened and ever-deepening public spirit and pride in our nation which devoted workers of all types in the Homeland are feeling with increasing intensity every day. All these must tend to chastened feelings, and to the adoption of higher standards of duty and work in everyday life, almost everywhere, throughout Great Britain. If we are to have memorials in future to noble pioneers and honoured workers, let all of us who have any influence resolve that every movement in this direction shall be zealously guarded against past abuses, which have amounted in some cases to a grave scandal.

We incline to the feeling that public opinion in the Provinces in regard to such matters has proved higher and more worthy than in the Metropolis. We published one of the most touching and convincing instances of this in The Hospital of August 18 last, page 402. In our issue of September 30 last, page 531, we gave a condensed account of a notable hospital chairman in the person of the late Sir Edward Wood, who, when overwhelmed with work and fully occupied with the responsible duties of Mayor of Leicester, consented at the age of sixty-four to become chairman of the Leicester Royal Infirmary, with the object of raising considerable funds to reconstruct and largely rebuild that institution. The inhabitants of Leicester have long made their mark on the nation by the exhibition of fearless independence, increasing public spirit, and the power to accomplish great things, quietly, with commendable ability and sound judgment. We may therefore feel certain that the movement inaugurated at the quarterly court of the Leicester Royal Infirmary on the 17th instant, to perpetuate the memory of Sir Edward Wood's notable work for his fellows in the town and county of Leicester, by rebuilding the south-west wing of the Infirmary, and so bring it up to date, will prove in every respect worthy of the great and good man whose work, as the chairman, Mr. T. Fielding Johnson, junior, properly insisted, has already proved of value to everyone in the county, and must continue to prove so to both rich and poor and to the medical profession. We have not space to set forth all the many good things Sir Edward Wood accomplished in his lifetime, as already recorded in The Hospital. The meeting was attended by leading representatives of all classes, from the Mayor downwards; the form of memorial selected, we have reason to feel, is the one Sir Edward would have most desired; and the required sum, £25,000, will, we are confident, be speedily forthcoming, and the important work put in hand without a moment's avoidable delay.

